# Effect of Different Types of Malocclusions on Maximum Bite Force in An Iranian Population: Effect of Malocclusions on Maximum Bite Force

**DOI:** 10.31661/gmj.v13iSP1.3601

**Published:** 2024-12-30

**Authors:** Ali Alamdari, Abbas Salehi Vaziri, Atefe Ahmadvand

**Affiliations:** ^1^ Faculty of Dentistry, Shahed University, Tehran, Iran; ^2^ Department of Orthodontics, Faculty of Dentistry, Shahed University, Tehran, Iran; ^3^ Department of Orthodontics, School of Dentistry, Shahid Beheshti University of Medical Sciences, Tehran, Iran

**Keywords:** Bite Force, Dental Occlusion, Malocclusion, Overbite, Angle Class III, Angle Class II, Angle Class I

## Abstract

**Background:**

Limited studies are available on how different malocclusion types and associated factors influence maximum bite force (MBF). Thus, this study aimed to assess the effect of the types of malocclusion, overbite, and overjet on MBF in an Iranian population.

**Materials and Methods:**

This cross-sectional, descriptive-analytical study involved 80 participants with no prior orthodontic treatments or jaw surgeries. The type of malocclusion of the participants was determined, and their overjet and overbite were measured. Their MBF was also measured by a strain gauge. The date were analyzed by one-way ANOVA and LSD test (alpha=0.05).

**Results:**

The mean MBF was 386.24 N in the study population (range 197 to 701 N). The mean MBF was 413.38 N, 381.11 N, and 165.33 N in Class I, Class II, and Class III patients, respectively (P0.05). Class III patients exhibited significantly lower MBF compared to the other groups (P0.05). Overjet and overbite had significant effects on the MBF (P0.05). The decreased overjet group showed significantly lower MBF compared to both the normal overjet (P=0.028) and increased overjet groups (P=0.011). Similarly, the deep bite (P=0.041) and edge-to-edge bite groups (P=0.02) had significantly lower MBF compared to the normal bite group.

**Conclusion:**

The results indicated that malocclusion type significantly influenced the MBF of the study participants, with Class III patients demonstrating notably lower MBF compared to other groups.

## Introduction

Occlusion and maximum bite force (MBF) are the main parameters to consider in the assessment of the efficiency of mastication [1]. Ideal treatment planning requires sufficient knowledge about muscle activity and its association with facial form. Assessment of MBF can aid orthodontists in the assessment of facial morphology and the type of mechanics used for treatment [2]. It can also aid in the diagnosis of maxillofacial disorders.

Occlusal force is defined as the force exerted on the teeth by the masticatory muscles [3]. MBF results from the coordinated action of various components of the masticatory system, including the teeth, muscles, and bones. The direct association of masticatory function and MBF has been previously confirmed [4].

Several independent factors affect the MBF, including the jaw morphology, dentition status, sensitivity of the temporomandibular joint and muscles of mastication, chewing habits, general and psychological health status, site of measurement of bite force, head position, patient’s fear of applying MBF, muscle thickness, age, and gender [5]. Gender is the most important factor affecting the MBF [3][6][7][8]. While a previous study found no significant gender difference in MBF [9], MBF tends to be higher in males due to greater muscle thickness and larger tooth size [3][6][7][8]. Morphological differences in the facial skeleton between males and females are also thought to contribute to these disparities [10].

Malocclusion, defined as misalignment of the teeth and improper maxillomandibular relationships, which has adverse esthetic and functional effects [6]. The type of malocclusion has also been suggested to affect the MBF [6][11]. A comprehensive knowledge of occlusion, mastication, and their effects on the growth and development of the masticatory muscles and facial structure, as well as the factors contributing to malocclusion, can aid in a better understanding of the complex nature of normal and abnormal occlusion. A thorough understanding of these physiological mechanisms facilitates the prediction of postoperative MBF changes in patients with different malocclusion types [6]. It appears that malocclusion decreases the MBF. Evidence shows that children with unilateral posterior crossbites and adults with anterior open bites usually have a lower MBF [6][12]. It should be noted that MBF has a significant association with the number of teeth present in the oral cavity [13][14][15]. Notably, the number of occlusal contacts has a more substantial impact on muscle function and MBF than the number of teeth [13]. It is believed that the number of occlusal contacts accounts for 10% to 20% of the variations in MBF, as adequate occlusal support and force distribution lead to improved muscle function and higher MBF. Since occlusion is a fundamental factor in the number of occlusal contacts, assessment of the MBF in different malocclusion types can better reveal the impact of this parameter on the quality of mastication. The lever action of the mandible and the function of the jaw’s elevator muscles result in greater occlusal force in the molar region than in the incisor area [16], which can be due to the higher number of occlusal contacts in the posterior region [13][16]. The number of occlusal contacts affects the mastication quality; therefore, the mastication efficiency is often lower in patients with malocclusion compared to those with normal occlusion [11].

Occlusion has both a static component, which refers to the relationship between the teeth and periodontium when the mandible is at rest, and a dynamic component, which addresses the inter-maxillary relationship during mandibular function. It is essential to assess both static and dynamic components of occlusion. Occlusal development and its impact on facial skeletal growth and muscle development should be further investigated to analyze the correlation of malocclusion with MBF [6].

The relationship of malocclusion with the function of the muscles of mastication remains a matter of controversy. Moreover, MBF varies across populations due to differences in facial and head morphology, occlusal patterns, and nutritional habits [17]. Given this variability, it is essential to examine MBF across diverse populations. Therefore, this study aims to evaluate the impact of types of malocclusion, overbite, and overjet on MBF in an Iranian population. The null hypothesis of the study posits that occlusion class, overbite, and overjet do not significantly affect MBF.

## Materials and Methods

**Table:** Table1. Measures of Central Dispersion for the MBF

**Parameter**	**Number**	**Minimum**	**Maximum**	**Mean**	**Std. error**	**Std. deviation**
**General**	80	197	70 **1**	386.24	13.42	120.07
**Male**	42	210	701	434.21	18.29	118.52
**Female**	38	197	580	332.21	15.98	98.54
**Round face**	31	220	701	400.77	21.78	121.29
**Oval face**	22	19 **7**	578	347.33	23.62	122.8
**Wide face**	27	21 **0**	605	413.5	22.89	107.36
**Class** **I**	45	216	701	413.38	18.09	121.37
**Class II**	26	197	574	381.11	21.96	112
**Class III**	9	210	314	165.33	12.66	37.99
**Decreased overjet**	14	21 **0**	476	337.64	21.09	78.92
**Normal overjet**	44	21 **6**	701	394.82	19.16	127.14
**Increased overjet**	22	19 **7**	580	400	26.41	123.86
**Open bite**	4	226	438	310.25	46.54	93.07
**Edge to edge bite**	4	278	325	308.75	10.50	21
**Normal bite**	63	197	701	403.92	15.21	120.74
**Deep bite**	9	220	57 **4**	330.67	39.61	118.83

This descriptive-analytical cross-sectional study was conducted on 80 participants who visited the Dental Clinic at the School of Dentistry, Shahed University. The study protocol was approved by the ethics committee of the university (IR.SHAHED.REC.1400.154).

### Eligibility Criteria

The inclusion criteria for participation in the study were:

• No history of orthodontic treatment or orthognathic surgery.

• No tooth loss or tooth wear that could influence occlusal function.

• No temporomandibular joint disorders or neurological conditions affecting the muscle force.

• Healthy individuals with no systemic diseases impacting occlusal function.

The exclusion criteria were:

• Presence of extensive restorations that could interfere with bite force measurements.

• Facial asymmetry or significant skeletal discrepancies.

• Any history of trauma or surgical interventions affecting the masticatory system.

• Systemic diseases affecting masticatory function (e.g., muscular dystrophy, neurological disorders).

### Sampling Method

The study employed a convenience sampling approach. Participants who met the inclusion criteria were consecutively enrolled from the pool of patients attending the dental clinic. A total of 80 participants (42 males, 38 females) were recruited, ensuring a relatively balanced gender distribution. This method of sampling was chosen to allow for the collection of a representative sample from the patient population attending the clinic within the study timeframe.

### Sample Size

The required sample size was determined using G-Power version 3.1.9.6. Based on a significance level of 0.05, a power of 0.80, and a medium effect size, it was calculated that 27 participants from each occlusion class and overjet group, and 20 participants from each overbite group, would be necessary. Recruitment continued until these target sample sizes were reached, and no additional participants were enrolled afterward.

### Data Collection

All participants provided written informed consent before the study, ensuring they understood the objectives and procedures involved.

### Demographic Data and Occlusion Classification

After obtaining consent, participants’ demographic information was recorded. The classification of occlusion was determined through clinical examination. According to Angle’s classification system:

• Class I: The mesiobuccal cusp of the maxillary first molar aligns with the groove of the mandibular first molar.

• Class II: The mesiobuccal cusp of the maxillary first molar is positioned distal to the mandibular first molar.

• Class III: The mesiobuccal cusp of the maxillary first molar is positioned mesial to the mandibular first molar.

### Overbite and Overjet Measurement

Overbite was measured as the vertical distance between the incisal edges of the upper and lower central incisors, perpendicular to the occlusal plane. Based on the measurements, participants were categorized into four overbite groups [18]:

• Open bite: Overbite≤ -1 mm

• Edge-to-edge overbite: Overbite>-1 mm but ≤ +1 mm

• Normal overbite: Overbite>+1 mm but≤ +4 mm

• Deep bite: Overbite>+4 mm

Overjet was measured as the horizontal distance between the incisal edge of the upper and lower central incisors, parallel to the occlusal plane. The following three overjet groups were used [18]:

• Decreased overjet: Overjet<2 mm

• Normal overjet: Overjet 2 to 3 mm

• Increased overjet: Overjet>3 mm

### Facial Measurements

Facial landmarks were measured using a digital caliper (Mitutoyo, Japan). Facial height was defined as the distance from the nasion to the gnathion, and facial width was defined as the inter-zygomatic distance. The facial pattern was calculated by dividing the facial height by facial width and multiplying by 100. Based on the resulting ratio, participants were categorized into three facial types:

• Wide

• Round

• Oval

### Measurement of Maximum Bite Force (MBF)

To measure MBF, participants were asked to sit upright with their head positioned in a natural resting position, ensuring the Frankfurt plane was parallel to the horizon. MBF was measured at both the right and left first molars using a strain gauge device (Zimec, with a 100 kg capacity). The device consisted of two metal plates attached to a load cell. When the patient applied force to the plates, the deformation was measured as a change in resistance, which was then displayed as a voltage reading (in Newtons, N). The plates were coated with a 4 mm thick condensation silicone impression material for comfort, and a new plastic wrap was used for each patient to avoid cross-contamination.

Participants were instructed to bite as forcefully as possible on the device for MBF measurement. Measurements were taken twice at each molar site, with a one-minute rest interval between measurements. If additional rest was required by the patient, a 5-minute interval was allowed. The highest recorded force at each molar site was used, and the average MBF between the right and left sides was calculated.

### Device Calibration

Before data collection, the device was calibrated using a known weight, ensuring accurate and consistent measurements. The calibration process involved placing specific weights on the sensor and confirming the displayed force. If any discrepancies were observed, the measurements were adjusted based on a calibration curve, achieving a high correlation coefficient (R²=0.9991) (Figure -1).

### Inter-examiner Reliability

To ensure the accuracy and reliability of the measurements, 20% of the MBF readings were repeated by a second trained examiner, and the results were compared. Any discrepancies between the two measurements were averaged and used for statistical analysis.

### Statistical Analysis

Data were analyzed using SPSS version 26 (SPSS Inc., IL, USA). Descriptive statistics (mean, standard deviation) were used to summarize the demographic and clinical characteristics of the sample. The primary analysis was conducted using one-way ANOVA to assess differences in MBF between the various occlusal classes, overbite, and overjet categories. Post hoc pairwise comparisons were performed using the LSD test to identify specific group differences. Multiple linear regression was employed to assess the relationship between occlusal parameters and MBF, adjusting for potential confounding factors such as gender and facial pattern. A significance level of 0.05 was considered for all statistical tests.

## Results

A total of 80 patients were evaluated, including 42 males (52.5%) and 38 females (47.5%). Of all, 38.8% had a round face, 27.5% had an oval face, and 33.8% had a wide face. Also, 56.3% had Class I, 32.5% had Class II, and 11.3% had Class III malocclusion. The frequency of decreased overjet, normal overjet, and increased overjet was 17.5%, 55%, and 27.5%, respectively. The distribution of overbite types was as follows: 5% had an open bite, 5% had an edge-to-edge bite, 78.8% had a normal bite, and 11.3% had a deep bite.

Table-1 presents the measures of central dispersion for the MBF.

### Effect of Type of Malocclusion on MBF

Pairwise comparisons of the classes of occlusion regarding MBF (Figure-2) revealed that the mean MBF in Class III patients was significantly lower than that in Class I (P=0.000) and Class II (P=0.001) patients. The difference in this regard between Class I and II patients was not significant (P=0.38787). The effect size was 0.2, indicating a moderate difference in MBF among different classes of occlusion.

### Effect of Overjet on MBF

ANOVA showed a significant effect of overjet on MBF (P=0.033). Pairwise comparisons by the LSD test (Figure-3) revealed that the MBF in the decreased overjet group was significantly lower than that in the normal overjet (P=0.028) and increased overjet (P=0.011) groups. The difference in MBF between the latter two groups was not significant (P=0.44). The effect size was 0.08, indicating a small difference in MBF among different types of overjet.

### Effect of Overbite on MBF

ANOVA showed a significant effect of overbite on MBF (P=0.030). Pairwise comparisons by the LSD test (Figure-4) revealed that the MBF in the deep bite (P=0.041) and edge-to-edge bite (P=0.02) groups was significantly lower than that in the normal bite group. No other significant differences were found. The effect size was 0.11, indicating a moderate difference in MBF among different types of overbite.

## Discussion

This study assessed the effect of type of malocclusion, overbite, and overjet on MBF in an Iranian population. The results showed significant effects of type of malocclusion, overjet, and overbite on MBF. Thus, the null hypothesis of the study was rejected.

The mean MBF in the study population was 386.24 N, which was lower than the MBF found in a previous study by Kadkhodazadeh et al. [19], which may be due to using a different device for measurement of MBF and not including the incisal bite force in the MBF [20].

The mean MBF in females was significantly lower than that in males in the present study, which was in agreement with previous findings [19][21][22]. This difference can likely be attributed to biological factors such as muscle mass and hormonal influences, which lead to greater masticatory muscle strength in males compared to females after puberty. The higher MBF in males may also be related to larger tooth size and more robust skeletal structure, which together provide a better foundation for generating greater bite force.

The MBF obtained in the present study was different from that reported by Sondang et al. [23] and Ferrario et al. [24], which can be due to racial differences of the study populations and subsequently different facial morphology and nutritional habits of the participants. The device and technique of measurement of MBF can also affect the results. A strain gauge was used for the measurement of MBF in the present study, which is extensively used for this purpose in the literature [21][25][26]. The present results showed that irrespective of other influential factors, the type of malocclusion had a significant effect on MBF, which was in contrast to the results of Sathyanarayana et al. [2], who showed that the maxillofacial sagittal morphology had no significant effect on MBF while vertical morphology had a significant effect on this parameter. The present study assessed the class of occlusion based on Angle’s classification, while Sathyanarayana et al. classified malocclusions according to skeletal facial morphology. This difference may explain the discrepancy between the two studies and suggests that class of occlusion may independently affect MBF, regardless of skeletal morphology. Alam and Alfawzan [6] and Araújo et al. [11] reported results consistent with the present findings and confirmed the effect of type of malocclusion on MBF, although Alam and Alfawzan [6] used a portable occlusal force gauge, which is more physiological than a strain gauge.

It appears that patients with different malocclusion types have a lower MBF than those with normal occlusion. English et al. [27] found results similar to the present findings regarding the effect of type of malocclusion on MBF and stated that malocclusion decreased the efficacy of mastication and MBF.

The significant difference in MBF between Class III and the other occlusal classes (I and II) aligns with previous studies that have suggested malocclusions may impair masticatory efficiency. Class III patients, who exhibit a more anteriorly positioned mandible, generally have fewer occlusal contacts compared to Class I and II patients. This reduced occlusal support likely results in decreased muscle function and a lower MBF, as the force generated during mastication is distributed over fewer teeth. This finding corroborates earlier studies by Alam and Alfawzan [6] and Araújo et al. [11], who found similar reductions in MBF in patients with malocclusion, especially Class III. Interestingly, no significant difference was observed between Class I and Class II malocclusion in this study, which contrasts with some earlier findings suggesting that Class II patients may have a lower MBF due to a more retrognathic jaw position and altered muscle mechanics [27][28][29][30]. This discrepancy may be due to the relatively small sample size and the inclusion of a diverse population with varying degrees of Class II malocclusion. It is also possible that compensatory mechanisms, such as the adaptation of the masticatory muscles, may mitigate any significant MBF differences between Class I and Class II patients. The present results also indicated significant effects of overjet and overbite on MBF. The MBF in the decreased overjet group was significantly lower than that in normal overjet and increased overjet groups, and the MBF in deep bite and edge-to-edge bite groups was significantly lower than that in the normal bite group. In some previous studies [13][31][32], the mean MBF was lower in patients with decreased overjet and open bite. Unlike the present study, Garner et al. [32] found no significant difference in MBF between patients with increased and decreased overjet, and only patients with normal overjet had a significantly higher MBF than other groups. This study’s results also align with those of Ow et al. [33]. Regarding overbite, the current study did not find a significant MBF difference between open bite and deep bite groups, which contrasts with findings by Turkistani et al. who reported a significant difference in their cohort [31]. This controversy can be due to differences in study populations since they evaluated European and American populations. Also, only a small percentage of patients had abnormal overbite (especially edge-to-edge and deep bite) in the present study, while the number of patients with different types of overbite was equal in their study. Alam and Alfawzan [6] reported significantly lower MBF in patients with decreased overbite than in patients with normal overbite and increased overbite.

### Limitations and Future Research

Despite offering valuable insights into how occlusal parameters influence MBF, this study has limitations. The sample size, particularly within smaller subgroups (e.g., Class III and edge-to-edge bite), may restrict the generalizability of the findings. Larger, more diverse studies are needed to confirm these results and investigate additional factors like muscle thickness and age that may affect MBF. Moreover, while strain gauges are commonly used to measure MBF, factors such as head position and sensor placement accuracy can affect their reliability. Future studies could incorporate more advanced methods, such as electromyography combined with bite force measurements, to examine individual muscle contributions to MBF and provide a more detailed understanding of the masticatory system.

## Conclusion

In conclusion, the findings of this study demonstrate that the class of occlusion, overjet, and overbite significantly affect MBF in an Iranian population. Class III malocclusion was associated with significantly lower MBF compared to other occlusal classes, while decreased overjet and deep bite also resulted in lower MBF. These findings underscore the importance of incorporating occlusal parameters in assessing masticatory function and planning orthodontic treatments. Future research should focus on exploring the underlying mechanisms that contribute to these variations in MBF and examine the impact of orthodontic treatment on MBF in patients with different occlusal conditions.

## Conflict of Interest

None.
